# Virginity testing: a systematic review

**DOI:** 10.1186/s12978-017-0319-0

**Published:** 2017-05-18

**Authors:** Rose McKeon Olson, Claudia García-Moreno

**Affiliations:** 1 University of Minnesota, 100 Church Street Southeast Minneapolis, Minneapolis, MN 55455 USA; 2 Department of Reproductive Health and Research, World Health Organization, 20 Ave Appia, Geneva, Switzerland 1227

**Keywords:** Virginity, Virginity testing, Hymen, Female, Gynecological examination

## Abstract

**Background:**

So-called virginity testing, also referred to as hymen, two-finger, or per vaginal examination, is the inspection of the female genitalia to assess if the examinee has had or has been habituated to sexual intercourse. This paper is the first systematic review of available evidence on the medical utility of virginity testing by hymen examination and its potential impacts on the examinee.

**Methods:**

Ten electronic databases and other sources for articles published in English were systematically searched from database inception until January 2017. Studies reporting on the medical utility or impact on the examinee of virginity testing were included. Evidence was summarized and assessed via a predesigned data abstraction form. Meta-analysis was not possible.

**Main Results:**

Seventeen of 1269 identified studies were included. Summary measures could not be computed due to study heterogeneity. Included studies found that hymen examination does not accurately or reliably predict virginity status. In addition, included studies reported that virginity testing could cause physical, psychological, and social harms to the examinee.

**Conclusions:**

Despite the lack of evidence of medical utility and the potential harms, health professionals in multiple settings continue to practice virginity testing, including when assessing for sexual assault. health professionals must be better informed and medical and other textbooks updated to reflect current medical knowledge. Countries should review their policies and move towards a banning of virginity testing.

**Electronic supplementary material:**

The online version of this article (doi:10.1186/s12978-017-0319-0) contains supplementary material, which is available to authorized users.

## Plain Language Summary

### Language: English

Virginity testing is a practice some communities use to detect which women or girls are ‘virgins’ (i.e. have not had sexual intercourse). People have different ways of trying to detect who are virgins. Some think you can tell by looking at the hymen (a piece of tissue that covers the vagina), while others think you can tell by looking at the size of the vagina. Communities often use the test to separate “pure” females from “impure” females. In some communities, only the “pure” females are to be married, have certain jobs, or be respected. This review searched ten different databases, and found 17 reports on virginity testing. We studied whether looking at the hymen can determine who is a ‘virgin’, and how the exam affects the girl or woman being tested. Our review found that virginity testing is not good at detecting who has not had sexual intercourse, and that it can hurt the person being tested – physically, mentally, and socially. Our hope is to make more people and countries aware of this to prevent harm to women and girls.

## Background

So-called virginity testing, also referred to as hymen, two-finger, or per vaginal examination, is the inspection of the female genitalia to determine if the individual has had or has been habituated to sexual intercourse [1]. The two most common techniques are inspection of the hymen for size or tears, and two-finger vaginal insertion to measure size of the introitus or laxity of the vaginal wall. Both techniques are performed under the belief that there is a specific appearance of genitalia that demonstrates habituation to sexual intercourse [1, 2]. The prevailing social rationale for testing is that an unmarried female’s virginity is indicative of her moral character and social value, whether in the context of marriage eligibility, sexual assault assessment, employment application, or otherwise [1, 2].

Virginity examinations are most commonly performed on unmarried females, often without consent or in situations where individuals are unable to give consent [1]. Depending on the region, the examiner may be a medical doctor, police officer, or community leader. Countries where this practice has been reported include Afghanistan, Bangladesh, Egypt, India, Indonesia, Iran, Jordan, Palestine, South Africa, Sri Lanka, Swaziland, Turkey, and Uganda [3–14]. Virginity testing is performed in various countries for reasons that vary by region. Certain communities in rural KwaZulu Natal in South Africa and Swaziland have performed virginity tests on school-aged girls with the aim to deter pre-marital sexual activity and reduce HIV prevalence [3, 4]. In India, the test has been part of the sexual assault assessment of female rape victims [9]. In Indonesia, the exam has been part of the application process for women to join the Indonesian police force [12, 13]. Due to increased globalization, reports of virginity testing are appearing in countries with no prior history, including Canada, Spain, Sweden, and the Netherlands [15]. Despite it being a long-standing tradition in some communities, formal assessments of the frequency of virginity testing are scarce. Thus, prevalence cannot be accurately described; however, anecdotes of its incidence occur in a variety of social settings in different countries.

The growing attention to eliminating sexual violence has raised awareness of the routine use of virginity testing in some settings [16]. This study was undertaken to systematically review all available published studies on virginity testing to determine its medical relevance and its impacts on the examinee. Ultimately, this review will inform the World Health Organization’s recommendations regarding virginity testing.

## Methods

**Fig. 1:**
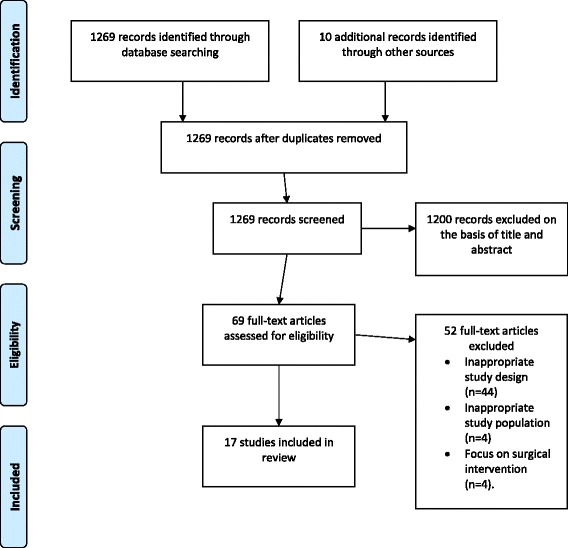
PRISMA flow diagram

This systematic review followed PRISMA guidelines (Fig. 1) [17]. The available literature on virginity testing was identified by searching ten electronic databases: Pubmed, the Cochrane Library, the Campbell Collaboration, SSRN, Regional Indexes of the WHO Global Health Libraries, Sage, Science Direct, Cambridge Press, Oxford Press, and Elsevier. Databases were searched for articles published in English from inception of the database until January 14, 2017. The search terms used were “virginity testing”, “virginity examination”, “hymen examination”, “two-finger testing”, and “per vaginal examination”. Multiple combinations of these search terms were used, with and without thesaurus and MeSH terms. (The search strategy is described in more detail in the Additional file 1). The protocol was not registered with a systematic review registry. Researchers in relevant fields were contacted for assistance in identifying studies. The reference lists of the identified studies were manually reviewed for additional citations.

The study population of interest was females who underwent any type of ‘virginity test’ and/or hymen examination. We did not enforce limitations on age, race/ethnicity, nationality, or other participant characteristics. Outcomes that were of interest in determining medical utility included physical exam findings of the female hymen (such as hymenal tear, perforation, or size of opening) that could indicate vaginal penetration, and the examiner’s ability to accurately and/or reliably identify hymenal features by physical exam. Outcomes that were of interest in determining impact on the examinee included personal or close-contact accounts of the effects of the virginity test on the examinee’s well-being (such as physical, psychological, and social consequences).

Two reviewers (Olson and García-Moreno) independently screened titles and abstracts and selected relevant studies for full text analysis. References of relevant articles were screened to find additional studies. RO then performed full text assessments, extracted data, and, in consultation with CGM, made decisions about study inclusion and exclusion. Any differences in opinion in the screening process, data extraction and in analysis were resolved through re-examination of the study and further discussion. If agreement had not been reached, the reviewers would have consulted a third reviewer.

Data were extracted using predesigned data extraction forms. The forms contained questions regarding study type, participant characteristics, role of examiner, method of examination, and outcomes measured. Data extracted from studies reporting on the impact of virginity testing on the examinee was synthesized with a thematic synthesis approach informed by the Cochrane Collaboration guidelines [18]. A spreadsheet was created of all the data extracted from these studies, and thematic analysis methods were used to develop broad themes.

The quality of each study was assessed using the grading system of the United States Preventive Services Task Force (USPSTF) [19, 20]. This grading system examines both study design and the internal validity of each study. (Additional information regarding the USPSTF grading criteria is provided in the Additional file 1). Internal validity measures how well the study was conducted, and a level of good, fair, or poor is assigned. Due to the lack of available research and presence of heterogeneity with respect to study design and aims, measures, and outcomes, a structured synthesis was undertaken, rather than a metaanalysis [21].

## Results

The search yielded 1269 articles, of which 69 full text articles were reviewed in full for eligibility. Of these, 17 met the inclusion criteria [4, 6, 14, 22–35]. All studies reporting primary data on the medical relevance and/or impact of virginity testing on examinee were included (*n* = 17). Studies with inappropriate study design were excluded (*n* = 44). This included editorials, opinions, and any study that did not report primary data on virginity testing and/or hymen examination. Studies with inappropriate study population were also excluded, including those that did not study females with a history of vaginal penetration (*n* = 4). Studies reporting on surgical interventions of the hymen not associated with virginity testing were excluded (*n* = 4). Ten studies reported on the medical utility of virginity testing and key findings are presented in Table 1 [22–31]. Eight studies reported on the impact of virginity testing on the examinee and key findings are presented in Table 2 and presented again in Table 3 by theme identified [4, 6, 14, 30, 32–35].

**Table 1:** Summary of included studies reporting on medical utility

Author, Year	Study design and population	Results	Quality of evidence
Berenson et al. 2000 [22]	Case-control study at two centers in United States (*n* = 392) Examiner(s): 1–2 physicians Age of examinees: 3–8 years Group 1: controls (*n* = 200) Group 2: females with history of penetration (*n* = 192)	2.5% of Group 2 had physical findings that differed from those found in Group 1 Only one hymenal feature difference was found between the two groups; a septate hymen was observed more often in Group 2 than Group 1 (4% vs 1%; *P* = .03)	II-2 Good
Kellog et al. 2004 [23]	Retrospective case review at one center in United States (*n* = 36) Examiner(s): 1 physician, 2 nurses Age of examinees: 12.3–17.8 years Study group: pregnant adolescents	22 participants (64%) had normal or nonspecific examination findings; 8 (22%) had inconclusive findings; 4 (8%) had suggestive findings; 2 (6%) had findings of definite evidence of penetrating trauma	II-2 Poor
Heger et al. 2002 [24]	5 year prospective study at one center in the United States (*n* = 2384) Examiner(s): 2 physicians Age of examinees: 3 months-14 years Study group: females who reported vaginal penetration (*n* = 957)	Abnormal examinations were reported in only 6% of females who reported vaginal penetration	II-2 Fair
Adams et al. 1994 [25]	Retrospective case review at one center in the United States (*n* = 236) Examiner(s): 1 staff of child abuse program Age of examinees: 8 months-17 years and 11 months Study group: girls who reported vaginal penetration/contact (*n* = 213)	Normal genital exam found in 59 cases (28%), non-specific exam in 104 cases (49%), and suspicious exam in 20 cases (9%) Size of hymenal opening of study group was 7.7 ± 2.6 mm and compared to published data on non-abused children of the same age 6.9 ± 2.2 mm	II-2 Fair
Berenson et al. 2002 [26]	Case-control study at two centers in the United States (*n* = 386) Examiner(s): 1–2 physicians Age of examinees: 3–8 years Group 1: controls (*n* = 197) Group 2: prepubertal females with history of penile or digital penetration (*n* = 189)	Group 2 had larger mean transverse hymen diameter than Group 1 when examined in the knee chest position but not supine position Hymenal orifice also increased with age No significant differences found between groups in size of vertical diameter, amount of tissue present inferiorly or laterally, or symmetry of hymen in either position	II-2 Good
Heppenstall-Heger et al. 2003 [27]	Prospective 10-year study at one center in United States (*n* = 94) Examiner(s): three pediatricians and three nurse practitioners Age of examinees: mean age 69.56 months (age range not specified) Study group: 75 female children with history of vaginal penetration or trauma	Hymenal injuries were found in 37 (49.3%) of 75 girls with history of vaginal penetration or trauma 15 girls (20%) persisted with significant genital findings (i.e., a transection of the hymen) In 80%, there was no hymenal irregularity	II-2 Fair
McCann et al. 2007 [28]	Retrospective case review at multiple centers in the United States (*n* = 239) Examiner(s): 1 physician and 2 nurses Age of examinees: 4 months-18 years Group 1: 113 prepubertal girls with history of vaginal penetration Group 2: 126 pubertal adolescents with history of vaginal penetration	The hymenal injuries in Group 1 and Group 2 all healed rapidly and frequently left little or no evidence of the previous trauma	II-2 Fair
Underhill et al. 1978 [29]	Case study at one center in the United States (*n* = 28) Examiner(s): 1–2 physicians Age of examinees: 15–48 years Study group: self-declared virgin females	Examination confirmed virginity in 58%, was inconclusive in 11% and unconfirmed in 31% of cases	II-3 Poor
Frank et al. 1999 [30]	Survey at one center in Turkey (*n* = 118) Examiner(s): forensic physicians Age of examinees: not specified Study group: forensic physicians	66% of respondents reported that their findings from at least one virginity examination contradicted a recent virginity examination of the same patient	III Fair
Dubow et al. 2005 [31]	Survey at one center in United States (*n* = 137) Examiner(s): pediatric chief residents Age of examinees: Not specified Study group: pediatric chief residents	64% correctly identified prepubertal hymen	III Fair

**Table 2:** Summary of included studies reporting on impact on examinee

Author, Year	Study design and population	Results	Quality of evidence
Leclerc-Madlala S 2003 [4]	Focus group interview in Durban, South Africa (*n* = 14) Examiner(s): elderly women Age of examinees: 13–18 years Study group: girls planning to attend upcoming virginity testing event	Girls reported fear that being “certified” a virgin would result in brothers, friends, or neighbors raping them Those who fail virginity tests are often expected to pay a fine for tainting the community and are excluded from certain employment	III Poor
Shalhoub-Kevorkian, N 2005 [6]	Interviews and focus groups in Jordan and Palestine (*n* = 41) Examiner(s): forensic medical doctors Age of Examinees: 21 years and younger Study group: 7 sexually assaulted women who had virginity testing, 17 police officers, 2 physicians, 7 prosecutors, 4 social workers, and 4 lawyers	5 of 7 interviewees described the harsh trauma and aftermath of the initial sexual assault and virginity exam afterward Focus group meetings showed women were extremely fearful and felt terrorized by virginity testing	III Fair
Robatjazi et al. 2015 [14]	In-depth semi-structured interviews in Iran (*n* = 15) Examiner(s): physicians and midwives Age of Examinees: not specified Study group: 11 physicians and 4 midwives who performed virginity tests	10 out of 11 physicians reported that virginity testing leads to psychological distress Most participants defined the following consequences of virginity testing: rejection, suicide, depression, weakened self-confidence, run-outs, divorce, and increased risk of diversion and abuse of girls	III Fair
Frank et al. 1999 [30]	Survey at one center in Turkey (*n* = 118) Examiner: forensic physicians Age of examinees: not specified Study group: forensic physicians	93% responded that virginity tests are psychologically traumatic for the patient, 64% believed they were a violation of privacy, and 60% believed they result in loss of examinee’s self-esteem	III Fair
Human Rights Watch 2010 [32]	Interviews in Delhi and Mumbai, India (*n* = 44) Examiner(s): gynecologists and forensic doctors Age of examinees: not specified Study group: direct contacts with virginity testing examinees including doctors, health rights activists, prosecutors, lawyers, and parents	The report documented the fear and re-traumatization of virginity testing on a rape victim Doctors were reported to have harmed the examinee during the test by aggravating existing injuries	III Poor
Human Rights Watch 2001 [33]	Interviews at eight public schools in three provinces of South Africa (*n* = 36) Examiner(s): Teachers and older women Age of examinees: 7–17 years Study group: girls who reported sexual violence at school, as well as teachers and counselors	Reported on the fear that a failed test will increase risk of abuse and discrimination In one case, a girl's relatives broke both her arms after she failed a virginity test	III Poor
Gursoy E, Vural G 2003 [34]	Survey in eight hospitals in Ankara, Turkey (*n* = 101) Examiner(s): nurses and midwives Age of examinees: not specified Study group: nurses and midwives	90% opposed and 10% supported virginity testing 62% agreed that a forced virginity exam might result in severe negative effects such as anxiety, depression, isolation from society, a dysfunctional sex life, guilt, worsened self-respect, and fear of death	III Fair
Leclerc-Madlala S. 2001 [35]	Observation, interviews, and focus groups in Durban, South Africa (sample size not specified) Examiner(s): elderly female relatives Age of examinees: 5–22 years Study group: key informants in virginity testing movement	Those who failed a virginity test were subject to name-calling and social exclusion Certified non-virgins were socially excluded, reporting that they will “spoil the bunch” and “cause the flowers of the nation to wilt”	III Poor

**Table 3:** Themes of impact on the examinee

Theme
Physical harm: Virginity tests resulted in physical harm to examinees. Reported incidents include injury caused by examiner, relatives, and examinee herself. Reports include examiner-induced aggravation of existing injuries, a failed test resulting in a relative breaking examinee’s arms, and completed suicide [6, 14, 32, 33].
Psychological harm: The psychological trauma of anticipating, experiencing, and recalling the virginity test was reported by examinees and witnesses. Included are reports of extreme fear and anxiety before the test, screaming, crying, and fainting during the test, and long-term effects of self-hatred, loss of self-esteem, violation of privacy, and re-victimization of previous sexual assault [6, 14, 30, 32, 34].
Social harm: The social effects of virginity testing were documented. Included are reports of a negative test bringing shame and dishonor to families and communities, social ostracism through marriage ineligibility and exclusion from jobs, and humiliation through name-calling. Positive tests also are reported to increase a virgin’s risk of sexual assault [4, 6, 35].

### Medical relevance

Ten studies reported on the medical relevance of hymen examination as a method to determine history of vaginal intercourse, the most common type of virginity testing [1]. The study characteristics and key findings are summarized in Table 1 [22–31]. The available research on this topic comes chiefly from physician examination of prepubertal and adolescent girls after sexual abuse allegations to determine if evidence of vaginal penetration existed. Seven of the included papers studied the accuracy of abnormal genital examinations as an indicator for history of vaginal penetration [22–28]. Abnormal genital exams included findings such as a hymenal transection, laceration, enlarged opening, or scar. Two studies reviewed physician’s accuracy in determination of virginity by exam [29, 30], and one study reported on pediatric chief residents’ ability to correctly identify the hymen by examination [31].

In a case-control study by Berenson et al. in the United States, genital features were compared between 192 girls with a history of vaginal penetration from sexual abuse and 200 who denied past penetration [22]. Presence or absence of 21 different hymenal or vulvar features was compared between the two groups, such as presence of hymenal tissue, transections, perforations, and notches. It was found that only 2.5% of physical exam findings were unique to the group with a history of penetration.

Kellog et al. studied a cohort in the United States with definitive evidence of previous vaginal penetration. In the study of 26 pregnant adolescents who reported sexual abuse, 22 participants (64%) had normal or nonspecific genital examination findings, eight (22%) had inconclusive findings, four (8%) had suggestive findings, and two (6%) had findings of definite evidence of vaginal penetration [23].

In one large cohort of 2384 children in the United States, 957 girls reported penetration from sexual abuse. Of these 957 girls, only 61 (6%) had abnormal genital examination findings [24]. The study’s parameters for abnormal examination included: “acute trauma, transections of the hymen that extended to the base of the hymen, scarring, sexually transmitted diseases, and positive forensics” [24].

In a retrospective case review in the United States of 213 girls who reported vaginal penetration/contact from sexual abuse, there was a normal genital exam in 59 cases (28%), non-specific findings in 104 cases (49%), and suspicious findings in 20 cases (9%) [25]. Size of hymenal opening was also measured on the study group (7.7 ± 2.6 mm) and compared to published data on non-abused children of the same age (6.9 ± 2.2 mm), and no significant difference was found in mean size [25].

Hymenal opening size was measured in a United States case-control study of 189 girls with, and 197 girls without, a history of penile or digital penetration from sexual abuse [26]. The former group had a larger mean transverse hymen diameter than the latter when examined in the knee-chest position but not supine position; hymenal orifice size also increased with age.

Healing of injuries to the hymen was reviewed in two studies, both in the contexts of sexual abuse allegations in the United States [27, 28]. In a study of 75 girls with a history of a vaginal penetration or trauma, injuries to the hymen were found in 37 cases (49.3%), significant genital findings (i.e., a transection of the hymen) were found in 15 girls (20%), and in the remaining 80%, there was no increase in the hymenal diameter or irregularity or narrowing of the hymen [27]. In a study of 113 prepubertal girls and 126 pubertal girls with previous penetration that reported on healing of injuries to the hymen, it was found that hymenal injuries in both groups healed rapidly and often left little or no evidence of previous trauma [28].

A 1978 study in the United States reported on the accuracy of physicians in confirming virginity through hymen examination [29]. Two gynecologists inspected the hymens of a cohort of 28 self-declared virgins. The physicians reported that examination of the hymen confirmed virginity in 16 cases (58%), was inconclusive in nine cases (31%), and uncertain in three cases (11%) [29].

In a study of forensic physicians in Turkey, 66% of respondents reported that their findings from at least one virginity exam contradicted a recent virginity exam of the same patient [30].

Lastly, a study examined physician knowledge of hymen anatomy [31]. In 2005, 137 United States pediatric chief residents were asked to identify the hymen on a photograph of pediatric anatomy; 64% were able to correctly identify the structure [31].

Quality of the ten studies reporting on the medical relevance of virginity testing was assessed according to USPSTF guidelines and is reported in Table 1. (Additional information regarding the USPSTF grading criteria is provided in the Additional file 1). The level of evidence ranged from level II-2 to level III. Seven studies were level II-2 [22–28], one study was level II-3 [29], and two studies were level III [30, 31]. The internal validity of the studies reporting on medical relevance ranged from good to poor. Two studies had good internal validity [22, 26], six had fair internal validity [24, 25, 27, 28, 30, 31], and two had poor interval validity [23, 29].

### Impact on examinee

Eight studies provided evidence on the effects of virginity testing on the examinee [4, 6, 14, 30, 32–35]. The study characteristics and key findings are summarized in Table 2. Included studies provided data on the experiences of the examinee and those who worked directly with the examinee (such as doctors, social workers, police officers, and lawyers). Six studies [4, 6, 14, 32, 33, 35] provided data from interviews and focus group discussions and two studies [30, 34] provided survey data from healthcare professionals. Three themes were constructed from the data: physical harm, psychological harm, and social harm. Themes are presented in Table 3, and expanded on below with relevant study findings and rationale of theme selection.

Physical harm of virginity examinees was reported in four studies [6, 14, 32, 33]. In a study of virginity testing in Palestine, a social worker present during her client’s virginity exam reported that, “the process was very painful, she was crying, screaming, holding my hands” [6]. Undergoing virginity exams also caused two of the social worker’s clients to become suicidal, with one reported attempted suicide [6]. When one examinee failed her virginity test, she was told that she would need to, “search for a way to save herself from the deadly consequences that awaited her” [6]. In a report of virginity testing in Iran, one medical examiner reported an exam that lead to death, “I told her that her hymen was not intact, and she said that she had done nothing. Then I heard she had committed suicide” [14]. A report on India’s two-finger test describes doctors who harmed the examinee during the test by aggravating existing injuries [32]. In South Africa, a report was made to Childline, a helpline that offers rape counseling, of an examinee’s relatives breaking both of her arms after she failed a virginity test [33].

Psychological harm was reported in five studies [6, 14, 30, 32, 34]. In a study of virginity testing in Palestine, focus group discussions revealed that women who underwent virginity testing were:

extremely fearful of and indeed felt terrorized by [the experience]. … Their feelings of fear and invasion were manifested in a variety of ways: by their refusal to sit on the examination chair, through crying, screaming, pushing, freezing-up, being silent, fainting, etc. [36]

One social worker described virginity exams as torture: “… I also felt it is so unfair to be sexually abused and then [have to] go through such a vicious process of torture” [6]. In depth interviews of medical professionals who performed virginity testing in Iran revealed that the virginity test resulted in the psychological distress of the examinee, causing “rejection, suicide, depression, and weakened self-confidence” [14]. A survey of forensic physicians in Turkey found that 93% of 118 respondents agreed that virginity tests are psychologically traumatic for the patient, 64% believed they were a violation of privacy, and 60% believed they result in loss of examinee’s self-esteem [30]. Interviews from a report on India’s two-finger test documented the fear and re-traumatization the examination causes [32]. In a study of 101 nurses and midwives in Turkey, 90% indicated they were opposed to virginity testing, and when asked why, nearly half agreed that they were opposed because the examinations are being done against the examinee’s will [34]. Sixty-two percent of the nurses and midwives also agreed that a forced virginity exam may result in severe negative effects such as anxiety, depression, isolation from society, a dysfunctional sex life, guilt, worsened self-respect, and fear of death [34].

Social harm was reported in three studies [4, 6, 35]. Leclerc-Madlala reported in a study in South Africa that those who fail virginity tests are often expected to pay a fine for tainting the community [4]. They are also excluded from certain employment opportunities, illustrated by one factory owner who stated that her various franchises throughout KwaZulu-Natal and the Eastern Cape had tested and selectively employed over 4000 virgins, which she believed was a service to the community and state [4]. Social harms of positive virginity tests were also noted. In a focus group interview of 14 girls who were planning to attend a virginity testing event, the girls reported that their primary concern was that being “certified” a virgin would result in brothers, friends, or neighbors raping them. They spoke of previous cases in which this had occurred in their community. Rape in this context was reported most likely to occur as a gang rape by several boys who needed to “teach her a lesson” and show her “what men are all about” [4]. A study in Palestine detailed one examinee’s fear of adverse social consequences [6]. She was afraid that a failed virginity test would result in loss of honor and social condemnation. The examinee stated, “I wanted to do [the examination]. I wanted to know if I lost my honor. I paid to learn that I lost my honor” [6]. In another study of South Africa’s KwaZulu-Natal province by Leclerc-Madlala, those who failed a virginity test were subject to name-calling and social exclusion [35]. One respondent referred to a girl who failed as a "rotten potato" who must be kept away from the ‘virgin girls’ because she will surely “spoil the bunch.” Another respondent noted that being in close proximity to a girl who failed the test would, “cause the flowers of the nation to wilt” [35].

Quality of the eight studies reporting on the impact of virginity testing on examinee was assessed according to USPSTF guidelines and is reported in Table 2. All eight studies were level III evidence [4, 6, 14, 30, 32–35]. The internal validity of the studies reporting on impact on examinee ranged from fair to poor. Four studies had fair internal validity [6, 14, 30, 34] and four had poor internal validity [4, 32, 33, 35].

## Discussion

The present review assessed 17 published studies on virginity testing, in particular its medical relevance and impact on the examinee.

The utility of hymen examination as a test for virginity was reviewed [22–31]. The studies indicated, as has been described in previous reviews, that the inspection of the hymen cannot give conclusive evidence of vaginal penetration or any other sexual history [36, 37]. Normal hymen examination findings are likely to occur in those with and without a history of vaginal penetration [22–30]. A hymen exam with abnormal findings is also inconclusive: abnormal hymenal features such a hymenal transection, laceration, enlarged opening, or scars are found in females with and without a history of sexual intercourse [22–30].

One hymenal feature commonly examined in virginity testing is hymenal opening size. Hymenal opening size also was found to be an unreliable test for vaginal penetration [25–27]. Hymen opening size varies with the method of examination, the position of the examinee, the cooperation and relaxation of the examinee, and the examinee’s age, weight, and height [25]. With regards to healing of hymenal injuries, it was found that most hymenal injuries heal rapidly and leave no evidence of previous trauma [26, 27].

Six studies reporting on medical utility included in the present review were limited by not having a control group [23–25, 27–29]. Studies without control groups make it difficult to interpret whether exam findings were accurately labeled as indicative or suspicious of vaginal penetration, as limited information is known about the appearance of the hymen after injury [22]. Another limitation is that most studies that reported on the medical utility of hymen examination were performed on females from the United States to determine if evidence existed after sexual abuse allegations, whereas most routine use of virginity tests has been reported outside of the United States and for the purpose of assessing moral or social value [1, 2]. The lack of data from outside the United States may affect the generalizability of results to females examined elsewhere. The ages of examinees of included studies were heterogeneous, with reported ages varying from 3 months to 48 years. Four studies combined females from different ages and stages of development [24, 25, 28, 29]. The heterogeneity in age groups may have caused observed differences to be due to differences in age, as it is known that hymenal features vary with age [23–25, 27–29]. Lastly, heterogeneity exists in regards to the knowledge and experience of examiner.

The search carried out for this review was comprehensive but did not include unpublished studies or studies in languages other than English, and thus there is potential to have missed relevant studies in other languages.

Another form of virginity testing is performed by insertion of two fingers into the vagina to examine its laxity [9]. This form of virginity testing was not included in the review of literature because the medical community has not considered vaginal laxity a clinical indicator of previous sexual intercourse. The vagina is a dynamic muscular canal that varies in size and shape depending on individual, developmental stage, physical position, and various hormonal factors such as sexual arousal and stress [38, 39]. However, there are reports that the so-called ‘finger testing’ has been used in countries like India to assess evidence for sexual assault [32]. It can also be found in forensic examination forms in some countries.

Studies of the effects of virginity testing on the examinee are also limited. Eight studies on the effects of virginity testing were identified and reviewed [4, 6, 14, 30, 32–35]. The review found that the virginity exam itself had resulted in physical harm of the examinee. This is supported by news reports from Turkey in which five by students attempted suicide by consuming rat poison to avoid undergoing the virginity test [7]. Virginity exams also commonly resulted in psychological trauma with long-lasting adverse effects, including but not limited to anxiety, depression, loss of self-esteem, and suicidal ideation. Health professionals also identified violation of privacy and autonomy as adverse effects [6, 34]. Lastly, virginity exams were reported to have an adverse social impact including social exclusion, perceived dishonor brought to family and community, employment discrimination, humiliation, and increased risk of sexual assault [4, 6, 35]. More research is needed to understand better the short and long term consequences of the virginity exam on the examinee.

## Conclusions and Recommendations

This review found that virginity examination, also known as two-finger, hymen, or per-vaginal examination, is not a useful clinical tool, and can be physically, psychologically, and socially devastating to the examinee. From a human rights perspective, virginity testing is a form of gender discrimination, as well as a violation of fundamental rights, and when carried out without consent, a form of sexual assault.

A gap exists between current medical evidence of virginity testing and medical education and training [9, 30, 33, 35]. Some forensic medical textbooks still include virginity testing as a standard procedure for assessment of sexual assault [38, 40–42]. Medical schools and public health professionals must update their textbooks, courses, and training to eliminate any recommendations of virginity testing, and educate others on the lack of scientific evidence for and possible harms of its use.

Governments, medical establishments, and health professional associations in all countries, even those with no history of virginity testing, should take the initiative to ban the use of virginity testing and create national guidelines for health professionals, public officials, and community leaders. More research is urgently needed to understand the regional and cultural rationales for virginity testing, and to develop more robust and efficacious education strategies that involve communities.

## Additional file

Additional file 1: Search strategy. United States Preventive Services Task Force Grading System [18]. (DOCX 21 kb)
