# Supplementary material for: Virginity testing: a systematic review
Source: Reprod Health. 2017 May 18;14:61. doi: 10.1186/s12978-017-0319-0 (PMC5437416; doi:10.1186/s12978-017-0319-0)
Supplement: Additional file 1: — Search strategy. United States Preventive Services Task Force Grading System [18]. (DOCX 21 kb) [file 12978_2017_319_MOESM1_ESM.docx]

Additional file 1: Search Strategy

Name of Searcher : Rose OLSON

Date of Search: 14/01/2017

Methodology:

(1) Medline/Pubmed was searched using both keywords and MeSH terms. The keyword search gave 1,023 results, and the MeSH search gave 157 results. When combined, Medline/Pubmed gave 1,026 results.

(2) Campbell, Cochrane, SSRN, and WHO Global Health Libraries were searched for published articles containing the word “virginity.” This gave 96 results.

(3) Databases including Sage, Science Direct, Cambridge, Oxford, and Elsevier were searched for articles that contained the terms “virginity test” or “virginity testing.” This gave 157 results.

(4) 10 additional sources were identified by contacting researchers in relevant fields

**Total results identified was 1,279. 10 duplicates were removed, resulting in 1,269 total records.**

1. Medline/PubMed Search Strategy

All years/dates, languages, and species were included.

(Virginity **OR** hymen **OR** “two finger” **OR** “per vaginal”)

**AND**

(test **OR** testing **OR** tests **OR** exam **OR** examination)

*Keyword Results:*

- *Concept 1: virginity/two-finger/hymen/per vaginal* 4,015
- *Concept 2: testing/exam*  4, 725,536

*Limits: none*

*When combing all concepts:* 1,023

_____________________________

("Sexual Abstinence"[Mesh] OR "Hymen"[Mesh] OR "two finger"[All Fields] OR "per vaginal"[All Fields])

**AND**

("Physical Examination"[Mesh] OR "Premarital Examinations"[Majr] OR "Gynecological Examination"[Mesh] OR "Mandatory Testing"[Majr] OR "Rape/diagnosis"[Mesh] OR "Forensic Sciences"[Mesh]

*MeSH Results:*

- *Concept 1: virginity/two-finger/hymen*  2,189
- *Concept 2: testing/exam*  1, 280,683

*Limits: none*

*When combing all concepts:* 157

*______________________________*

Total Pubmed Results when combining Keyword and MeSH results: 1,026

*______________________________*

2. Campbell Collaboration Library, Cochrane Library, SSRN, and the WHO Regional Indexes of the Global Health Libraries Search Strategy

All years/dates, languages, and species were included.

The Campbell Collaboration Library, the Cochrane Library, the Social Science Research Network (SSRN), and the WHO Global Health Library Databases (including LILACS (AMRO/PAHO), AIM (AFRO), IMEMR (EMRO), and WPRIM (WPRO)) were searched for journals that contained the word “virginity” in all text.

*Results:*

- *Campbell Collaboration Library: 0*
- *Cochrane Library: 6*
- *SSRN: 25*
- *Global Health Libraries: 65*
  - *LILACS (AMBRO/PAHO): 58*
  - *AIM (AFRO): 3*
  - *IMEMR (EMRO): 3*
  - *WPRIM (WPRO): 1*
- *Total: 96*

*________________________________*

3. Sage Publication Online, Science Direct, Cambridge University Press Journals, Oxford University Press Journals, and Elsevier Search Strategy

All years/dates, languages, and species were included.

Publication databases were also searched for journals that contained the terms “virginity testing” or “virginity test” or “virginity test” anywhere in the article.

*Results:*

- *Sage Publication Online: 62*
- *Science Direct: 59*
- *Cambridge University Press Journals: 19*
- *Oxford University Press Journals: 17*
- *Elsevier Science: 0*
- *Total: 157*

_____________________________________

In summary:

Total records identified = 1279

Duplicates = 10

Total records after duplicates removed = 1269

United States Preventive Services Task Force Grading System

Levels of Evidence According to the United States Preventive Services Task Force [32, 33]

| Level | Origin of Evidence |
| --- | --- |
| I | Evidence obtained from at least one properly designed randomized controlled trial |
| II-1 | Evidence obtained from well-designed controlled trials with randomization |
| II-2 | Evidence obtained from well-designed cohort or case-control analytic studies, preferably from more than one center or research group |
| II-3 | Evidence obtained from multiple time series with or without intervention  Marked results in uncontrolled experiments might also be regarded as this type of evidence |
| III | Opinions of respected authorities on the basis of clinical experience, descriptive studies, or reports of expert communities |

Criteria for evaluating the internal validity of studies according to the United States Preventive Services Task Force [32]

| Study Design | Criteria |
| --- | --- |
| Systematic Reviews | ▪ Comprehensiveness of sources and search strategy used  ▪ Standard appraisal of included studies  ▪ Validity of conclusions  ▪ Recentness and relevance |
| Case-Control Studies | ▪ Accurate ascertainment of cases  ▪ Non-biased selection of cases and controls with exclusion criteria applied equally to both  ▪ Response rate  ▪ Diagnostic testing procedures applied equally to each group  ▪ Appropriate attention to potential confounding variables |
| RCTs and Cohort Studies | ▪ For RCTs: adequate randomization, including concealment and whether potential confounders were distributed equally among groups  ▪ For cohort studies: consideration of potential confounders with either restriction or measurement for adjustment in the analysis; consideration of inception cohorts  ▪ Maintenance of comparable groups (includes attrition, crossovers, adherence, or contamination)  ▪ Important differential loss to follow-up or overall high loss to follow-up  ▪ Measurements: equal, reliable, and valid (includes masking of outcome assessment)  ▪ Clear definition of interventions  ▪ All important outcomes considered  ▪ Analysis: adjustment for potential confounders for cohort studies, or intention-to-treat analysis for RCTs |
| Diagnostic Accuracy Studies | ▪ Screening test relevant, available for primary care, adequately described  ▪ Study uses a credible reference standard, performed irrespective of test results  ▪ Reference standard interpreted independently of screening test  ▪ Handles indeterminate results in a reasonable manner  ▪ Range of patients included in study  ▪ Sample size  ▪ Administration of reliable screening test |

Abbreviation: RCT, randomized controlled trial.
